# Curative effects of sophorolipid on physical wounds: In vitro and in vivo studies

**DOI:** 10.1002/vms3.481

**Published:** 2021-03-25

**Authors:** Min‐Jin Kwak, Min‐Young Park, Jonggun Kim, Hanbae Lee, Kwang‐Youn Whang

**Affiliations:** ^1^ Department of Biotechnology Korea University Seoul Republic of Korea; ^2^ Pathway Intermediates Seoul Republic of Korea

**Keywords:** early‐weaning syndrome, glycolipid emulsifier, gut remodelling, intestinal microflora, short‐chain fatty acid

## Abstract

Early‐weaning syndrome is harmful to animals because an effect on growth in the early‐stage of life generally determines the overall growth rate. Sophorolipid (SPL), a surface‐active glycolipid compound, has been shown to exhibit antimicrobial activity and stimulate cell proliferation. Thus, in vitro and in vivo studies were conducted to evaluate the potential of SPL on the gut turnover after the wound. The in vitro experiment with HT‐29 cells showed the increased proliferation with increasing gene levels of collagenase‐1 and matrilysin‐1. Next, the 16‐day in vivo experiment was conducted with thirty rats (14‐day‐old), and the allocation was performed according to their body weight (BW) into three treatments: control diet (CON), 48 ppm of oxytetracycline‐supplemented diet (OTC) and 10 ppm of SPL‐supplemented diet (SPL). Dietary SPL accelerates the growth of rats in overall periods, and intestinal permeability was lower in SPL at day 16. Villus:crypt ratio and the goblet cell count were also higher in SPL than in CON at day 8. Caecal *Streptococcus* spp. were significantly reduced with dietary SPL and OTC at day 8 and 16, and total short‐chain fatty acid, acetate and butyrate levels were increased in the SPL at day 8. In conclusion, these data demonstrated that SPL could improve gut remodelling potential and modulate the gut environments, resulted in acceleration of post‐weaning growth. Therefore, SPL could have a potential as a feed additive aimed at promoting repair system after wound in animal's gut.

## Graphical abstract

Sophorolipid upregulated the wound healing process. Gut defence system was improved by dietary sophorolipid. Caecal populations of microflora and short‐chain fatty acid were modulated by oral sophorolipid intake.

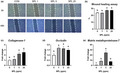

## 1 INTRODUCTION

Growth retardation after weaning is one of the irritating experience in young animals due to physiological, environmental and social changes occurred by separation from their mother. Because this retardation can influence the overall growth of the animal until the time of slaughter, the livestock industry has been searching for improved methods to relieve the stress from weaning by housing, nutrition and management aspects (Campbell et al., 2013).

One such strategy is the use of antibiotic dietary additives as antibiotic growth promoters, which can have a significant effect on growth acceleration and feed efficiency improvement (Wegener, 2003). However, the use of antibiotics had been warned by World Health Organization (WHO) in 2014 because livestock can serve as a reservoir of antibiotic‐resistant bacteria (Bates et al., 1994). Thus, the livestock industry is seeking an alternative to antibiotics. Previous studies have demonstrated that various feed additives have the potential to be a replacement of antibiotics (Markovi et al., 2009; Wang et al., 2010), but none of these have proven cost‐effective in their growth‐promoting effects or modulation of gut microbial populations (Niewold, 2007). A novel and eco‐friendly alternative feed additive is therefore needed to substitute for antibiotics.

Recently, the antimicrobial properties of bio‐surfactants have attracted attention owing to their low toxicity and high bio‐degradability. Of particular interest is sophorolipid (SPL), a glycolipid bio‐surfactant produced by non‐pathogenic yeast species such as *Candida bombicola* (Cho et al., 1999). Sophorolipid has lower toxicity and higher bio‐degradability than other surfactants (Desai & Banat, 1997), and it displays many unique biological properties including antimicrobial activity, immune modulation, stimulation of skin dermal fibroblasts and collagen production (Concaix, 2003; Gross & Shah, 2003; Maingault, 1999). These properties suggest that SPL could have a potential to improve animal health and growth after weaning. Therefore, this study aimed to evaluate the beneficial effects of SPL on early‐weaning syndrome.

## 2 MATERIALS AND METHODS

### 2.1 In vitro study (wound healing assay)

HT‐29 cell line, human colorectal adenocarcinoma cell line, was achieved from the Korean Cell Line Bank. Cells were sustained in Roswell Park Memorial Institute 1640 medium with 10% heat‐inactivated fetal bovine serum (thawed at 4°C, then incubated at 65°C for 30 min) and 1% penicillin and streptomycin at 37°C and 5% CO_2_ in a humidified chamber. Afterwards, a wound healing assay (cell migration test) was performed as previously described (Rodriquez et al., 2005), using three doses of SPL (1, 5 and 25 µg/ml). Briefly, HT‐29 cells were seeded onto 6‐well plates and grew until they reached 100% confluency. Randomly selected sites of the cell monolayer were then manually wounded by scratching with a pipette tip, and the cells were further incubated with/without SPL. Cells were photographed immediately after wounding (0 hr) and after 48 hr of incubation.

### 2.2 mRNA analysis by quantitative real‐time PCR

Trizol® (Invitrogen) was used to extract total RNA according to the manufacturer's procedure. And High‐capacity cDNA Reverse Transcription kit (Applied Biosystems) was used to synthesize cDNA. Amplications of target genes were determined using a RealHelix^TM^ Premier qPCR Kit (NanoHelix) with a StepOnePlus Real‐Time PCR System (Applied Biosystems). Primers are listed in Table 1. The 2^−ΔΔCT^ method was used to quantify the relative mRNA expression levels.

**TABLE 1:** Oligonucleotide primers used in HT‐29 cells and jejunal qRT‐PCR analysis

Gene name	Sequence (forward, reverse)
In vitro experiment	
GAPDH	5′‐CGGAGTCAACGGATTTGGTCGTAT‐3′
	5′‐AGCCTTCTCCATGGTGGTGAAGAC‐3′
MMP‐1	5′‐CCAGGCAGCTTAACAAAGGC‐3′
	5′‐CCCAGCACTCACTTTACGGT‐3′
MMP‐7	5′‐TGGCCTACCTATAACTGGAA‐3′
	5′‐TCCCTAGACTGCTACCATCC‐3′
In vivo experiment	
Total bacteria	5′‐GCAGGCCTAACACATGCAAGTC‐3′
	5′‐CTGCTGCCTCCCGTAGGAGT‐3′
*Escherichia coli*	5′‐CATGCCGCGTGTATGAAGAA‐3′
	5′‐CGGGTAACGTCAATGAGCAAA‐3′
*Streptococcus* spp.	5′‐GTACAGTTGCTTCAGGACGTATC‐3′
	5′‐ACGTTCGATTTCATCACGTTG‐3′
*Salmonella* spp.	5′‐AACGTGTTTCCGTGCGTAAT‐3′
	5′‐TCCATCAAATTAGCGGAGGC‐3′

Abbreviations: GAPDH, glyceraldehyde 3‐phosphate dehydrogenase; MMP‐1, collagenase‐1; MMP‐7, matrilysin‐1.

### 2.3 Animal and experimental diets

All of the works related to animal was approved by the Korea University Institutional Animal Care and Use Committee (KU‐IACUC). And all procedures for animal were conducted in accordance with the standard guidelines and protocols of Korea University (Approval number: KUIACUC‐2020‐0097). Thirty early‐weaned male Sprague Dawley rats (14‐days old) were used in a 16‐day experiment (average body weight: 23.95 g). Rats were randomly allocated into three experimental treatment groups according to their initial body weight, with ten replications. The control diet was NIH‐41 diet and its composition is presented in Table 2. Dietary treatments were as follows: (a) control diet, CON group; (b) control diet + 48 ppm oxytetracycline (OTC), OTC group; (c) control diet + 10 ppm SPL, SPL group. Feed was provided in powdered form and freely allowed to rats. All rats were housed in a clean and sanitary environment.

**TABLE 2:** Ingredients and calculated composition of NIH‐41 diet

Ingredients, g/kg	
Ground whole hard wheat	34.90
Ground #2 yellow corn	21.00
Ground whole oats	10.00
Wheat middlings	10.00
Fish meal	9.00
Soy oil	2.00
Soybean meal	5.00
Alfalfa meal	2.00
Corn gluten meal	2.00
Dicalcium phosphate	1.50
Yeast‐Brewers	1.00
Premixes^a^	0.60
Grounded limestone	0.50
Salt	0.50
Calculated composition	
Crude protein, g/kg	180.0
Crude fat, g/kg	50.0
Crude fibre, g/kg	50.0
Ash, g/kg	80.0
Calcium, g/kg	10.0
Total phosphorous, g/kg	8.5
Lysine, g/kg	8.5
Methionine, g/kg	3.5

^a^ Premixes: vitamin A, 14,500 IU/kg; vitamin D_3_, 4,600 IU/kg; vitamin K, 2.8 mg/kg; α‐tocopheryl acetate, 20 IU/kg; choline, 560 mg/kg; folic acid, 2.2 mg/kg; niacin, 30 mg/kg; pantothenic acid, 18 mg/kg; riboflavin, 6.6 mg/kg; thiamin, 10 mg/kg; vitamin B_12_, 0.058 mg/kg; pyridoxine, 1.7 mg/kg; biotin, 0.113 mg/kg; cobalt, 0.4 mg/kg; copper, 4 mg/kg; iron, 60 mg/kg; magnesium, 400 mg/kg; manganese, 100 mg/kg; zinc, 10 mg/kg; iodine, 1.5 mg/kg.

### 2.4 Experimental procedures and sample collection

This experiment was conducted in two phases. Phase I (day 1–8) estimated the restoration effect and phase II (day 9–16) investigated growth promoting effect. Body weight (BW) and feed intake were recorded on day 0, 8 and 16, and average daily gain (ADG), average daily feed intake (ADFI) and feed efficiency (FE) were calculated. Half of rats were euthanized at day 8, and the other half were euthanized at day 16. Blood, jejunum and caecal content samples were collected. Blood samples were collected into EDTA vacutainer tubes (BD vacutainer) from heart after anaesthesia. Plasma samples were obtained by centrifugation (3,000 *g*, 30 min, 4°C). Jejunum and caecal content samples were obtained and frozen immediately with liquid nitrogen, then stored at −80°C until further analysis. Also, jejunum section samples were fixed in 4% formalin for histological analysis.

### 2.5 Gut permeability test

Fluorescein isothiocyanate‐dextran 4 (FD4; TdB Consultancy) was used to assess paracellular uptake. Food and water were removed from the cages 6 hr before euthanasia, and rats were given FD4 (0.44 g/kg BW) by oral gavage needle 4 hr before euthanasia. The optical density of FD4 was measured in each sample using a spectrophotofluorometer with excitation at 485 nm and an emission at 535 nm.

### 2.6 Histological analysis of jejunum

All fixed jejunum samples were embedded in paraffin using a Miles Scientific 4586 Tissue‐Tek Dispensing Console (Miles Scientific), and 5 µm sections were obtained using a Rotary Microtome CUT 5062 (SLEE MAINZ). Sections were stained with Haematoxylin‐Alcian Blue‐Sirius Red staining according to standard protocols.

### 2.7 Caecal bacterial populations by quantitative real‐time PCR

Genomic DNA from caecal contents was extracted by a Quick‐DNA^TM^ Faecal/Soil Microbe Microprep kit (Zymo Research) according to the manufacturer's protocol. Target bacteria were amplified and assayed using a RealHelix^TM^ Premier qPCR Kit (NanoHelix) with a StepOnePlus Real‐Time PCR System (Applied Biosystems). Table 1 showed the primers list. The concentration of total bacteria was used as a housekeeping control. The 2^−ΔΔCT^ method was used to quantify the relative bacteria levels.

### 2.8 Gas chromatography‐mass spectrometry for caecal short‐chain fatty acid

The concentration of caecal short‐chain fatty acid (SCFA; acetate, propionate and butyrate) was measured using gas chromatography‐mass spectrometry (GC‐MS) (Furusawa et al., 2013). SCFA concentrations were quantified with peak areas and calculated with standards curves.

### 2.9 Statistical analysis

Data were analysed using the analysis of variance procedure (ANOVA) with Statistical Analysis System 9.4 (SAS 9.4, 2011). Significant differences between treatment means were determined using Duncan's multiple‐range tests. *p* < .05 was defined as significant.

## 3 RESULTS

### 3.1 In vitro experiment

**FIGURE 1:**
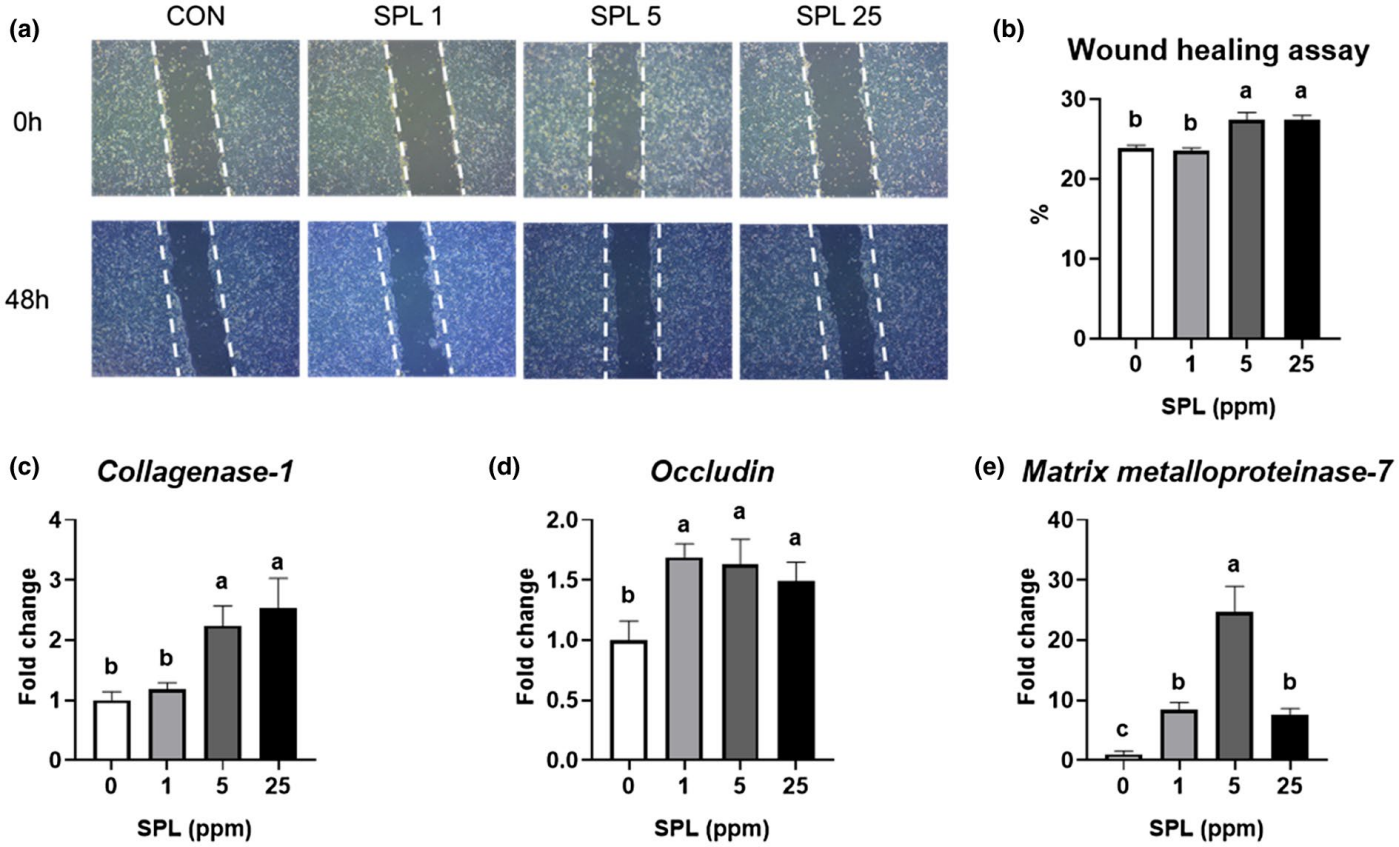
Representative images from the wound healing assay at 0 hr and 48 hr (a) and percentage of enclosed area after wounding of HT‐29 cells (b). mRNA expression levels of collagenase‐1 (c), occludin (d) and Matrix metalloproteinase‐7 (e) after wounding of HT‐29 cells. Data are expressed as mean ± SE. ^a,b,c^Different superscripts in the same series indicate statistically significant differences (*p* < .05). SPL, sophorolipid. CON, no treatment; SPL1, 1 ppm of SPL treatment; SPL5, 5 ppm of SPL treatment; SPL25, 25 ppm of SPL treatment

Results and representative images of the wound healing assay 48 hr after SPL treatment are shown (Figure 1a,b). After 48 hr, the largest cell coverage was seen in 5 and 25 ppm of SPL‐supplemented dishes (*p* < .05). To elucidate the SPL's specific mode of action, the expression of genes related to wound healing and structural physiology was investigated using qRT‐PCR. Collagenase‐1 (MMP‐1) expression was significantly increased (*p* < .05) by 5 and 25 ppm SPL supplementation (Figure 1c). And all dosages of SPL significantly increased occludin and matrilysin‐1 (MMP‐7) expression (Figure 1d,e).

### 3.2 In vivo experiment

Average BW at day 0 was 23.97 g and there was no difference between treatment groups. At day 8, the average BW of rats was similar among dietary treatment groups; however, the average BWs of animals in the OTC and SPL were numerically increased at day 16, respectively, compared to the CON group. In addition, the ADG was higher in the OTC and SPL than in the CON over the whole experimental period. The ADFI of the SPL was numerically increased when compared to the CON and OTC, respectively, from day 0 to day 16. Consequently, FE was significantly highest (*p* < .05) in the rats fed OTC‐supplemented diet (Table 3).

**TABLE 3:** Growth performance of rats fed experimental diet^a^, ^b^

Treatment	CON	OTC	SPL	*SEM*	*p*‐value
BW, kg					
Day 1	23.95	23.97	23.93	0.562	1.000
Day 8	36.62	38.74	38.58	1.234	.723
Day 16	74.72	84.32	86.01	3.707	.424
ADG, kg/day					
Day 1–8	1.58	1.85	1.75	0.103	.536
Day 8–16	4.52	5.11	5.55	0.253	.318
Day 1–16	3.12	3.64	3.78	0.191	.351
ADFI, kg/day					
Day 1–8	3.14	3.13	3.53	0.127	.527
Day 8–16	8.25	9.20	10.05	0.448	.350
Day 1–16	5.82	6.30	6.92	0.289	.425
FE					
Day 1–8	0.50	0.57	0.49	0.017	.118
Day 8–16	0.55	0.56	0.55	0.003	.428
Day 1–16	0.53^b^	0.58^a^	0.55^a^, ^b^	0.007	.013

^a^ Abbreviations: ADG, average daily gain; ADFI, average daily feed intake; BW, body weight; CON, control; FE, feed efficiency; OTC, oxytetracyclin; *SEM*, standard error of the mean; SPL, sophorolipid.

^b^ CON, NIH‐41 diet; OTC, 48 g/kg of oxytetracycline‐supplemented diet; SPL, 10 g/kg of sophorolipid‐supplemented diet.

**FIGURE 2:**
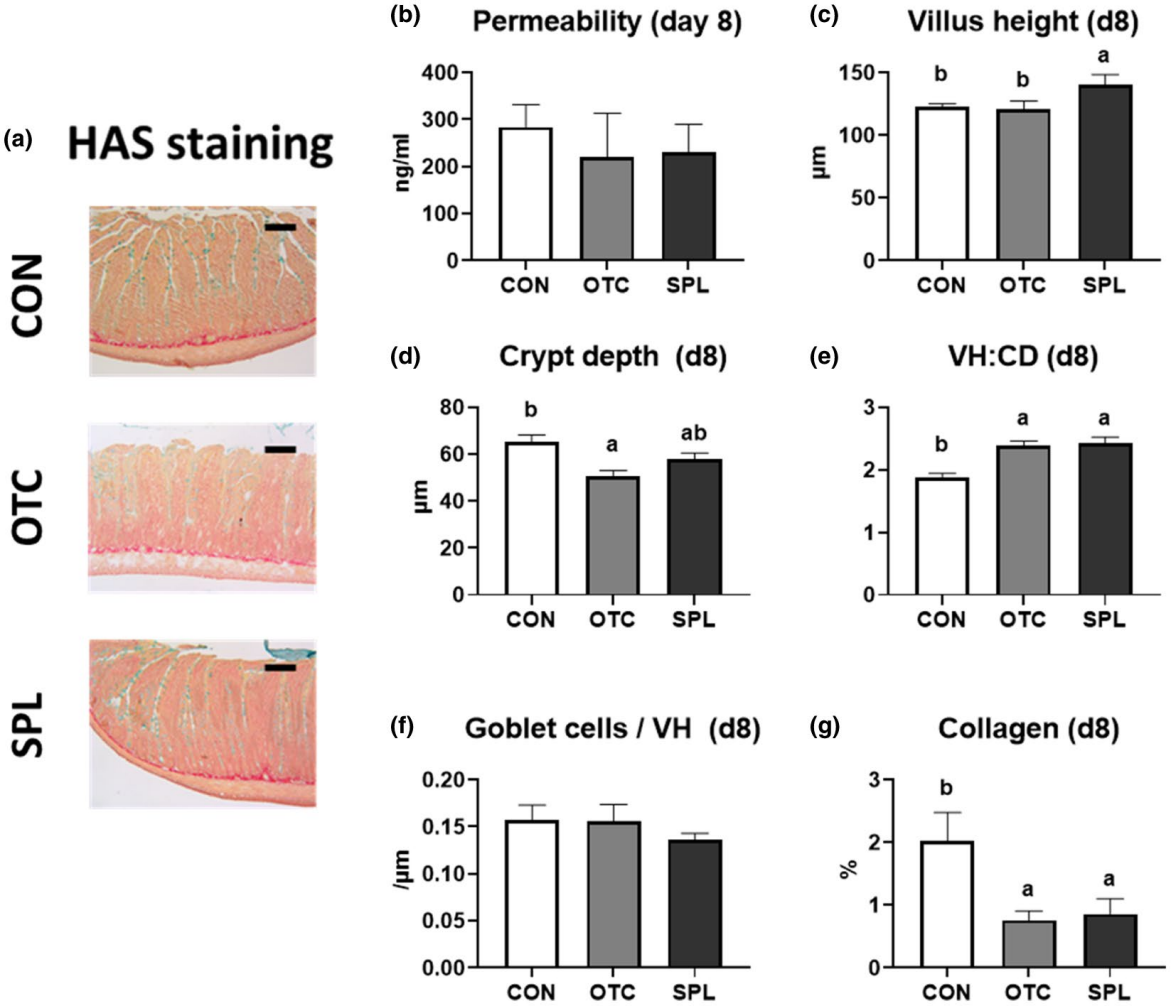
Representative pictures of jejunum stained by Haematoxylin‐Alcian Blue‐Sirius Red methods (a), paracellular intestinal permeability (b), villus height (c), crypt depth (d), villus height: crypt depth (e), goblet cells per 1 µm villus height (f), and the portion of collagen (g) at day 8. CD, crypt depth; CON, control; HAS staining, Haematoxylin‐Alcian Bule‐Sirius Red staining; OTC, oxytetracycline; SPL, sophorolipid; VH, villus height. Control diet (CON), 48 ppm of OTC‐supplemented diet (OTC) and 10 ppm of SPL‐supplemented diet (SPL). Data are expressed as mean ± SE. Bar = 250 µm. ^a,b^Different superscripts in the same series indicate statistically significant differences (*p* < .05)

**FIGURE 3:**
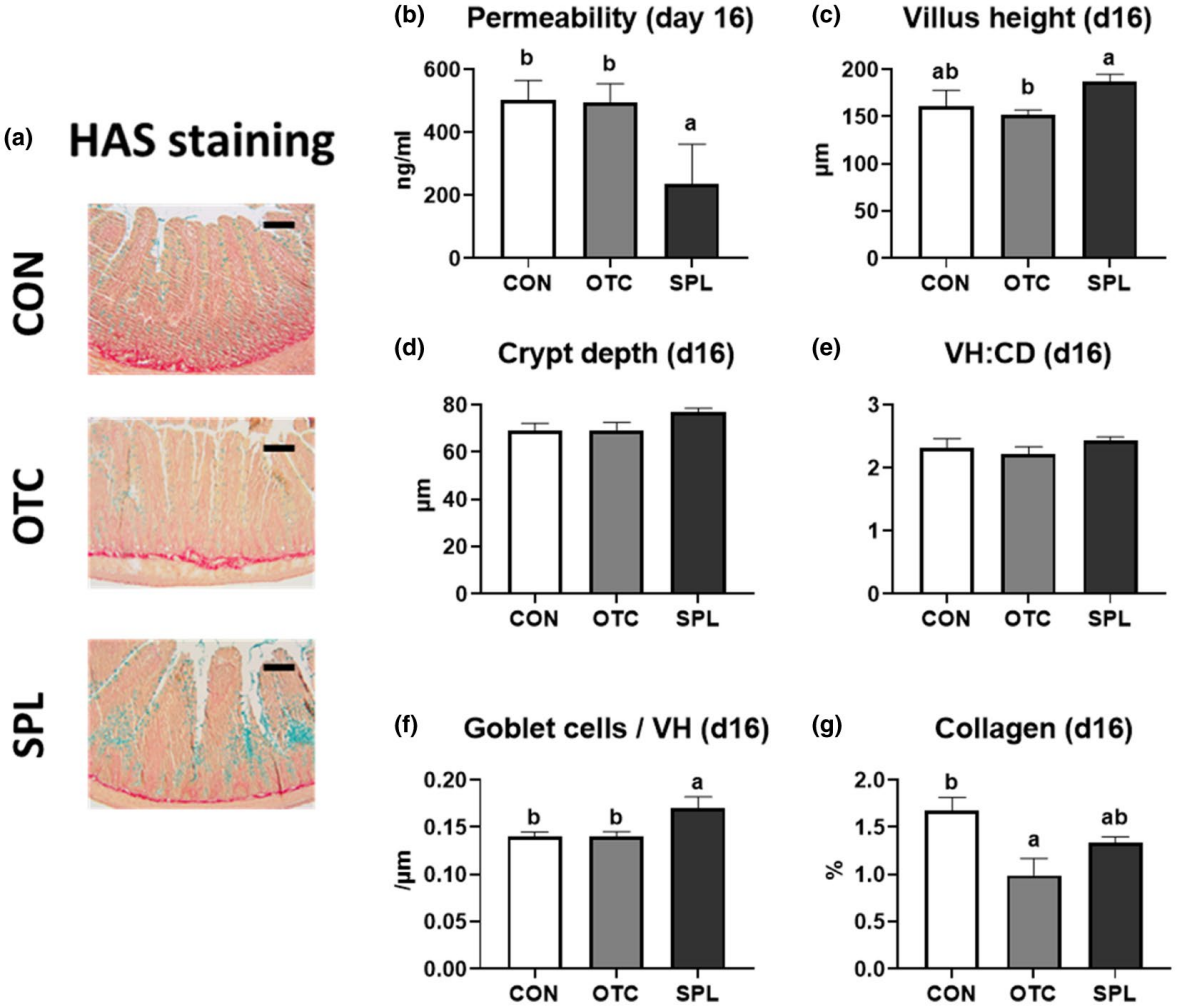
Representative pictures of jejunum stained by Haematoxylin‐Alcian Blue‐Sirius Red methods (a), paracellular intestinal permeability (b), villus height (c), crypt depth (d), villus height: crypt depth (e), goblet cells per 1 µm villus height (f), and the portion of collagen (g) at day 16. CD, crypt depth; CON, control; HAS staining, Haematoxylin‐Alcian Bule‐Sirius Red staining; OTC, oxytetracycline; SPL, sophorolipid; VH, villus height. Control diet (CON), 48 ppm of OTC‐supplemented diet (OTC) and 10 ppm of SPL‐supplemented diet (SPL). Data are expressed as mean ± SE. Bar = 250 µm. ^a,b^Different superscripts in the same series indicate statistically significant differences (*p* < .05)

The representative pictures and the results of jejunum at day 8 and day 16 were shown in Figures 2 and 3. There was no change in intestinal permeability with dietary supplementation at day 8 (Figure 2b), but dietary SPL significantly decreased (*p* < .05) paracellular permeability compared to CON and OTC at day 16 (Figure 3b). Villus height of rat fed 10 ppm of SPL‐supplemented diet was significantly lengthened (*p* < .05) after 8 and 16 days when compared to other treatments (Figures 2c and 3c). Crypt depth of rats' jejunum was significantly decreased (*p* < .05) in OTC compared to CON at day 8, however, that was not changed at day 16 (Figures 2d and 3d). Consequently, the villus: crypt ratios in the OTC and SPL were significantly higher (*p* < .05) when compared to the CON at only day 8, not day 16 (Figures 2e and 3e). Rats fed a SPL‐supplemented diet had not changed the number of goblet cells per 1 µm of villus at day 8; however, it was significantly increased (*p* < .05) by dietary SPL supplementation compared to the CON group at day 16 (Figures 2f and 3f). On the other hands, the portions of collagen in OTC and SPL were significantly reduced (*p* < .05) at day 8, and collagen in SPL was increased at day 16 (Figures 2g and 3g).

**FIGURE 4:**
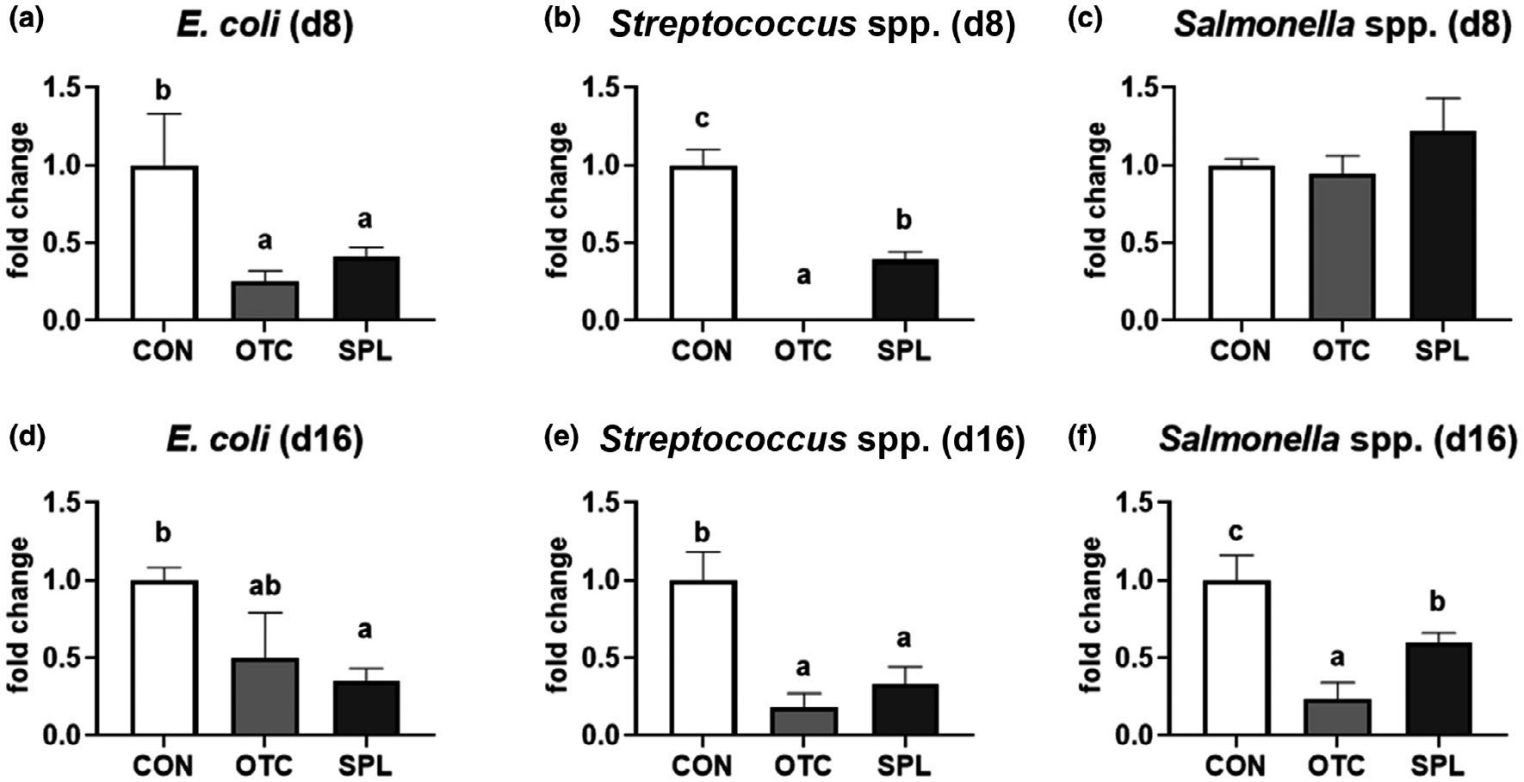
Relative populations of caecal pathogenic bacteria, including *Escherichia coli* (a, d), *Streptococcus* spp. (b, e) and *Salmonella* spp. (c, f) of early‐weaned rats fed experimental diets for 8 and 16 days. CON, control; OTC, oxytetracycline; SPL, sophorolipid. Control diet (CON), 48 ppm of OTC‐supplemented diet (OTC) and 10 ppm of SPL‐supplemented diet (SPL). Data expressed as mean ± SE. ^a,b,c^Different superscripts in the same series indicate statistically significant differences (*p* < .05)

To investigate the antimicrobial effects of SPL, caecal pathogenic bacterial populations (*Escherichia coli*, *Streptococcus* spp. and *Salmonella* spp.) were assessed using qRT‐PCR (Figure 4). At day 8 and 16, the populations of *E. coli* were reduced (*p* < .05) in OTC and SPL compared to CON (Figure 4a,d). Similarly, dietary SPL and OTC supplementation dramatically reduced (*p* < .05) *Streptococcus* spp. at day 8 and day 16, compared to the CON (Figure 4b,e). Dietary OTC and SPL supplementation also caused a significant reduction (*p* < .05) in *Salmonella* spp. compared to the CON at day 16; however, this effect was not found at day 8 (Figure 4c,f).

**FIGURE 5:**
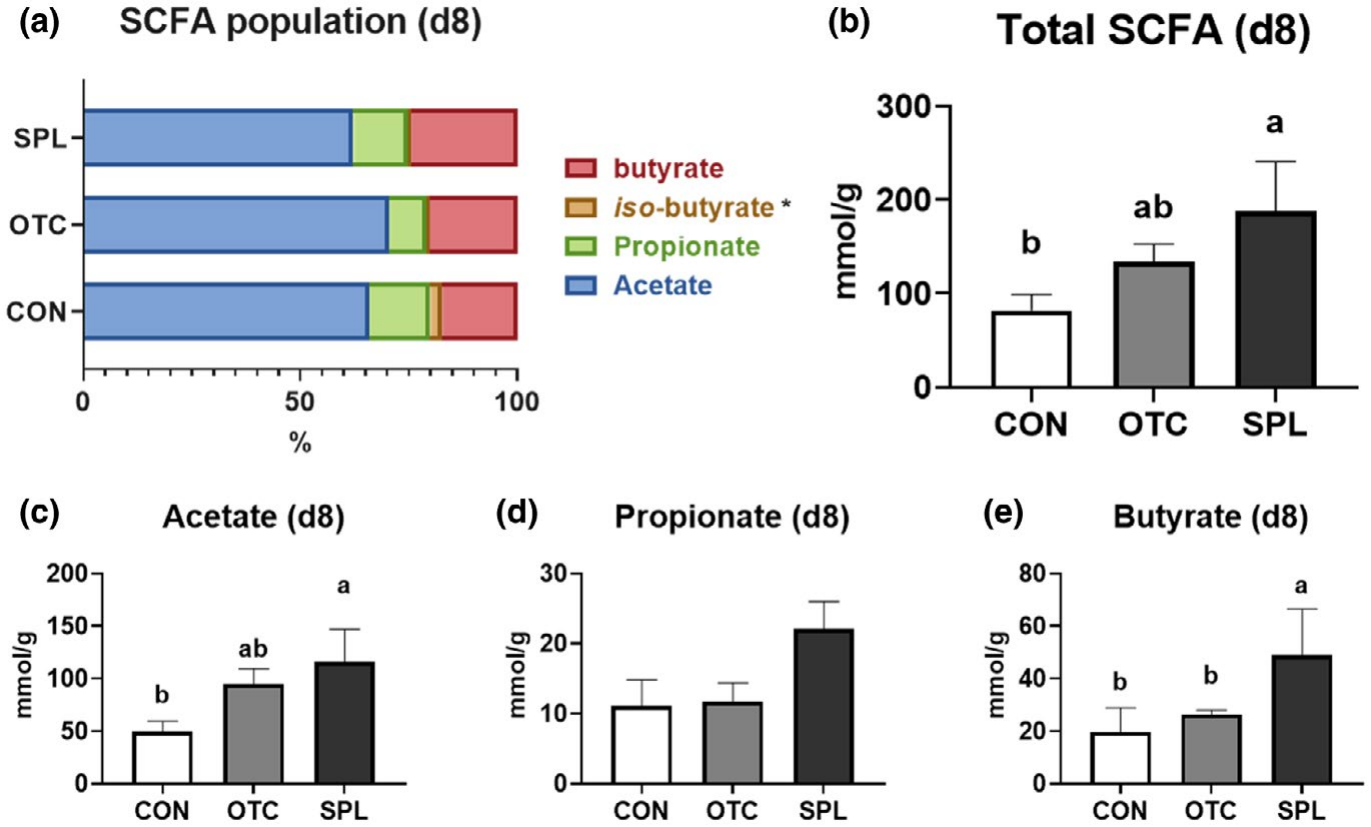
Relative short‐chain fatty acid concentration (a), total short‐chain fatty acid concentration (b), and the portion of acetate (c), propionate (d) and butyrate (e) of rats fed experimental diets for 8 days. CON, control; OTC, oxytetracycline; SCFA, short‐chain fatty acid; SPL, sophorolipid. Control diet (CON), 48 ppm of OTC‐supplemented diet (OTC) and 10 ppm of SPL‐supplemented diet (SPL). Data expressed as mean ± SE. ^a,b^Different superscripts in the same series indicate statistically significant differences (*p* < .05)

**FIGURE 6:**
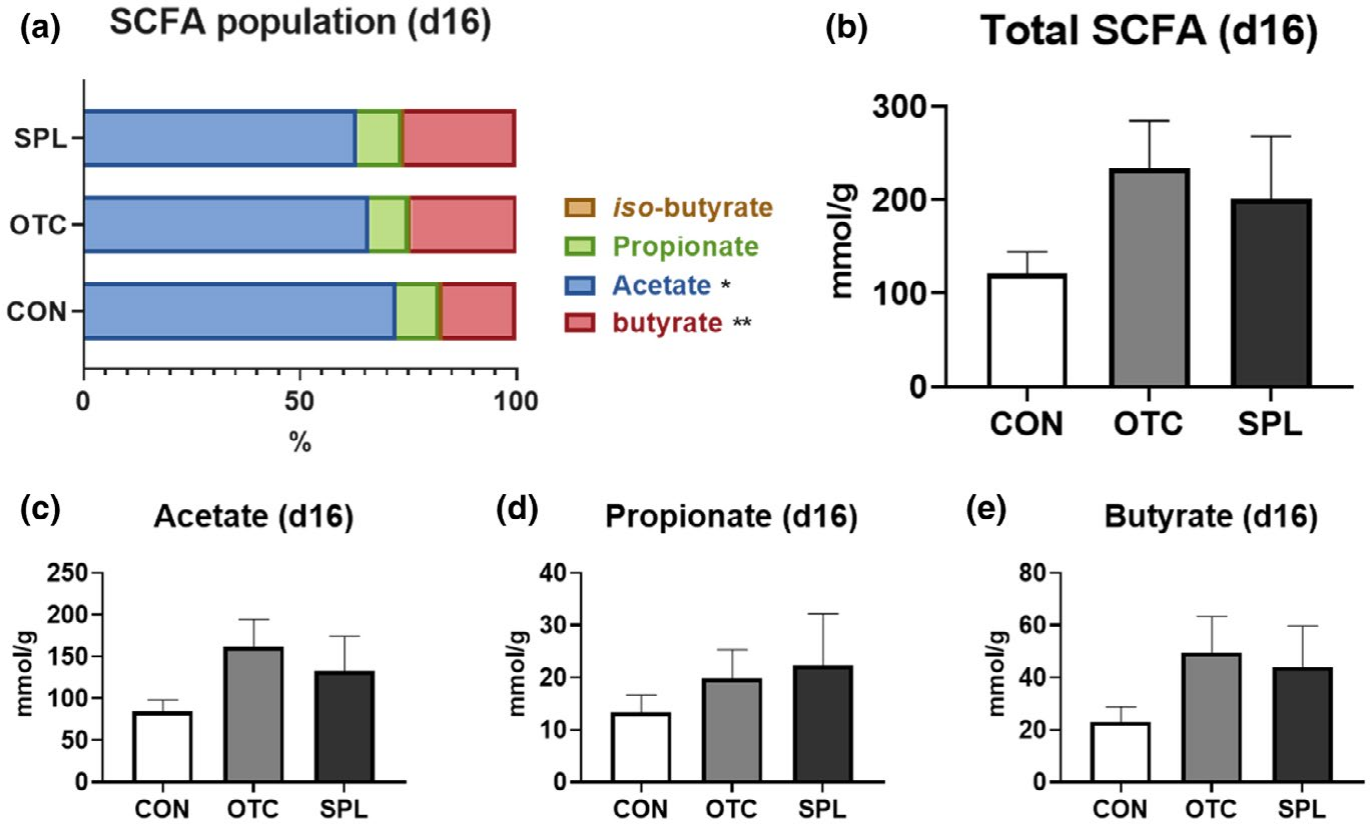
Relative short‐chain fatty acid concentration (a), total short‐chain fatty acid concentration (b), and the portion of acetate (c), propionate (d) and butyrate (e) of rats fed experimental diets for 16 days. CON, control; OTC, oxytetracycline; SCFA, short‐chain fatty acid; SPL, sophorolipid. Control diet (CON), 48 ppm of OTC‐supplemented diet (OTC) and 10 ppm of SPL‐supplemented diet (SPL). Data expressed as mean ± SE

Caecal SCFA concentration was measured to elucidate the specific mechanisms underlying modulation of the caecal bacterial population at day 8 and 16 (Figures 5 and 6). The concentrations of acetate, propionate (*p* = .135) and butyrate of rats in SPL group were significantly increased (*p* < .05) compared to CON at day 8 (Figure 5c–e), resulted in the significant increment (*p* < .05) of total SCFA concentration in rats fed SPL‐supplemented diet (Figure 5a). However, the concentration of all kind of SCFA was not changed by dietary SPL at day 16 (Figure 6). Dietary SPL supplementation resulted in an increase in total SCFA concentration when compared to CON in restoration phase.

## 4 DISCUSSION

Weaning causes severe physical and psychosocial stress in young animals, both as a result of separation from their mother as well as changes in the form of their feed. The stress destroys intestinal morphological, microbiological and immunological environments and leads to dysfunction such as diarrhoea (van Beers‐Schreurs et al., 1992). Therefore, researchers in the livestock industry have begun to investigate ways to accelerate gut restoration during the post‐weaning period in terms of nutritional and pharmacological aspects (Dirkzwager et al., 2005). One strategy being tested is dietary supplementation with antibiotics, which can alter the gut microbiota and help to maintain intestinal health and function (Adjiri‐Awere & van Lunen, 2005; Taras et al., 2007). However, the livestock feed industry has been seeking alternatives to antibiotics because they can induce bacterial resistance by providing a reservoir for antibiotic‐resistant genes (Woolhouse et al., 2015). In this study, the restoration effect of SPL was investigated in wound treatment using an in vitro assay. We also assessed the antimicrobial and therapeutic effects of SPL in early‐weaned rats and evaluated whether SPL could substitute for the dietary efficacy of antibiotics.

The effects of glycolipid‐type bio‐surfactants on various cell types have been reported (Jezierska et al., 2018); however, the little information about relationship between SPL and intestinal epithelial cell was provided. In this study, we showed that SPL accelerated proliferation and migration in an in vitro wound model using HT‐29 cells. Because rapid cell migration and proliferation play a key role in gut recovery following injury (Jung et al., 2009), our results suggest that SPL may be able to aid in the acceleration of intestinal wound healing. To elucidate the mode of action of SPL, we analysed the gene expression levels related to the wound healing process. Matrix metalloproteinases (MMP) are an enzyme family, which contains Zn ion and depends on Ca ion (Nagase & Woessner, 1999). And their functions are specifically related to angiogenesis, wound healing, tissue remodelling, cell migration and homing of inflammatory cells to the wound site (Parks, 1999). Particularly, MMP‐1 is important in epithelial cell migration, while MMP‐7 is involved in epithelial closure and membrane remodelling during gut wound repair (Pilcher et al., 1997; Saarialho‐Kere et al., 1996). In the current study, the proper dosages of SPL treatment showed upregulating MMP‐1 and MMP‐7 expressions, suggesting that SPL treatment could improve intestinal healing by affecting epithelial cell migration and remodelling.

The single epithelial cell layer of the digestive tract is the widest area of contact between outside and inside of the body. Hence, intestinal barrier function is important because it is the organ that encounters the most external substances, including pathogens, antigens, toxins, as well as nutrients (Moeser et al., 2017). For this reason, we investigated gut paracellular permeability and morphology to assess leakiness of the epithelium. Results from in vivo study suggest that dietary SPL may improve jejunal permeability and morphology. Thus, together with the results of in vitro experiment showing its proliferation‐accelerating effects, it is possible that SPL could aid in restoration of the damaged intestine that occurs after weaning. Consistent with our results, Maingault demonstrated that a sophorolipidic compound could activate macrophages, and thus serve as a healing agent in wounds (Maingault, 1999). At the same time, a thick layer of mucus, secreted by goblet cells, encloses the surface of epithelial cells and builds a barrier to extraneous toxic materials (Cornick et al., 2015), thus having a role as the first line of protection, particularly counter to pathogenic bacteria (McCracken & Lorenz, 2001). We found that dietary SPL supplementation dramatically increased mucus secretion in the jejunum. In summary, dietary supplementation with SPL could vastly improve the gut defence environment by promoting intestinal wound healing and increasing mucus production.

Next, we analysed caecal pathogenic bacteria to address the possibility that SPL could replace the antimicrobial efficacy of antibiotic. Our results suggest that dietary SPL supplementation may attenuate the adaptation of gut pathogenic bacteria, including *E. coli*, *Streptococcus* spp. and *Salmonella* spp. These findings are consistent with other reports, especially one showing that SPL produced by lauryl alcohol had potential anti‐bacterial effects on both *E. coli* and *Streptococcus aureus* (Pulate et al., 2013). However, further studies will be needed to investigate more species within the intestinal microbiota.

The role of SCFA and its link to the gut microbiome has been extensively studied because SCFA is the end‐product of gut microbiota fermentation with dietary non‐starch polysaccharides which cannot be digested by animals (Le Poul et al., 2003). In this study, we found that the concentration of all SCFA was increased in rats given SPL‐supplemented diet, and suggest that the gut microflora modulated by dietary SPL may produce larger amounts of SCFA including acetate, propionate and butyrate. Subsequently, it also seemed that dietary SPL was able to modulate the gut microbiota to digest larger amounts of non‐starch polysaccharides. In accordance with our study, Xu et al demonstrated that SPL increases SCFA production by increasing the abundance of hydrolytic microbes and SCFA producers in waste activated sludge, while Maingault suggested that a sophorolipidic compound could act as a fibrinolytic agent (Maingault, 1999; Xu et al., 2019). Conversely, the SCFA modulation in gut might be able to regulate hepatic lipid metabolism and adipose tissue stabilization (Nishina & Freedland, 1990). In particular, acetate was found to promote lipogenesis and cholesterol genesis in the liver, while both were inhibited by propionate (Demigne et al., 1995). It is thought that the ratio of acetate to propionate could be crucial to the relationship between liver and caecal SCFA concentrations (Morrison & Preston, 2016). Taken together, our results demonstrated that SPL as a dietary additive could modulate the gut microbiome and SCFA levels, and may also work to regulate general lipid metabolism in the host.

## 5 CONCLUSION

In conclusion, SPL appears to improve jejunal permeability and the gut defence system, as well as the caecal microbiota and SCFA levels, which in turn could aid in growth acceleration after weaning. The present study represents the first trial using SPL as a feed additive, and suggests that SPL could be a novel nutritional strategy aimed at overcoming early‐weaning syndrome.

## CONFLICT OF INTEREST

The authors declare no conflicts of interest.

## AUTHOR CONTRIBUTION

**Min‐Jin Kwak:** Data curation; Formal analysis; Investigation; Validation; Visualization; Writing‐original draft; Writing‐review & editing. **Min‐Young Park:** Data curation; Investigation; Methodology; Validation. **Jonggun Kim:** Methodology. **Hanbae Lee:** Funding acquisition; Resources. **Kwang‐Youn Whang:** Conceptualization; Funding acquisition; Methodology; Project administration; Supervision.

## ANIMAL WELFARE STATEMENT

The authors confirm that the ethical policies of the journal, as noted on the journal's author guidelines page, have been adhered to and the appropriate ethical review committee approval has been received. The authors confirm that they have followed EU standards for the protection of animals used for scientific purposes. All of the works related to animal were approved by the Korea University (KU) Institutional Animal Care and Use Committee (IACUC). All the procedures for animal were conducted in accordance with the standard guidelines and protocols of KU.

### PEER REVIEW

The peer review history for this article is available at https://publons.com/publon/10.1002/vms3.481.
